# ORATOR2-Studie – randomisierter Vergleich einer primären Operation oder primären Strahlentherapie bei HPV-positivem Oropharynxkarzinom im Kontext einer Therapiedeeskalation

**DOI:** 10.1007/s00066-022-01987-2

**Published:** 2022-08-01

**Authors:** Alexander Fabian, Nils H. Nicolay, David Krug

**Affiliations:** 1 Klinik für Strahlenheilkunde, Universitätsklinikum Freiburg, Robert-Koch-Str. 3, 79106 Freiburg, Deutschland; 2 Klinik für Strahlentherapie, Universitätsklinikum Schleswig-Holstein (UKSH), Campus Kiel, Arnold-Heller-Str. 3, 24105 Kiel, Deutschland

## Hintergrund

Die Inzidenz HPV-assoziierter Oropharynxkarzinome steigt. Gleichzeitig sind HPV-assoziierte Oropharynxkarzinome im Vergleich zu HPV-negativen Karzinomen insbesondere bei Nichtrauchern mit einer deutlich besseren Prognose assoziiert. Die Frage nach einer Therapiedeeskalation bei PatientInnen mit HPV-assoziiertem Oropharynxkarzinom ist daher aktueller Gegenstand intensiver Forschung mit unterschiedlichen Ansätzen und dem Ziel, die therapiebedingte Morbidität zu senken, ohne den Therapieerfolg zu beeinträchtigen. Dieser Frage widmeten sich Palma und Kollegen in der ORATOR2-Studie.

## Patienten und Methodik

Diese multizentrische, 1:1 randomisierte Phase-II-Studie schloss PatientInnen mit HPV-assoziiertem Oropharynxkarzinom (T1–2, N0–2 ohne Hinweis für kapselüberschreitendes Wachstum [ECE], M0) ein. Initial war die Rekrutierung von 140 PatientInnen geplant.

Die Randomisierung erfolgte stratifiziert nach Raucherstatus in einen Arm mit deeskalierter primärer Radio(chemo)therapie (RT-Arm) oder einen Arm mit primärer transoraler Chirurgie (TOS; OP-Arm). Der RT-Arm erhielt intensitätsmoduliert 60 Gy auf makroskopische Tumoranteile, 54 Gy auf Hochrisiko- und 48 Gy auf Niedrigrisikolymphabflusswege in 30 Fraktionen. Bei alleiniger Radiotherapie war eine Akzelerierung mit 6 Fraktionen pro Woche zulässig. Sofern ein Lymphknoten größer als 3 cm war oder mehr als ein suspekter Lymphknoten vorlag, erfolgte eine simultane Chemosensibilisierung mit Cisplatin (40 mg/m^2^, 1 ×/Woche, 6 Zyklen). Der OP-Arm erhielt eine transorale laser- oder robotergestützte Resektion (Sicherheitssaum 1 cm) mit risikoadaptierter uni- oder bilateraler Neck-Dissektion. Eine adjuvante Radiotherapie war indiziert bei ECE, positiven oder knappen (< 3 mm) Resektionsrändern, mehr als einem betroffenen Lymphknoten, pT3/4 oder lymphovaskulärer Invasion. PatientInnen mit ECE oder positivem Resektionsrand erhielten intensitätsmoduliert 60 Gy auf die Region des ECE bzw. des positiven Resektionsrands, 54 Gy auf die OP-Region einschließlich Primarius und dissezierter Lymphknotenlevel sowie 48 Gy auf die Niedrigrisikolymphabflusswege in 30 Fraktionen. Die übrigen PatientInnen mit Indikation zur Adjuvanz erhielten intensitätsmoduliert 50 Gy auf die OP-Region einschließlich Primarius und dissezierter Lymphknotenlevel sowie 45 Gy auf die Niedrigrisikolymphabflusswege in 25 Fraktionen. Eine simultane Chemosensibilisierung sollte nur bei makroskopischem Resttumor und Erfüllung der Kriterien für eine Chemosensibilisierung des RT-Arms erfolgen. ECE und/oder positive Resektionsränder allein waren keine Indikation für eine simultane Chemosensibilisierung. In beiden Armen wurde bei Tonsillenkarzinomen eine unilaterale Radiotherapie empfohlen, sofern der Tumor mehr als 1 cm vom Zungengrund und weichen Gaumen entfernt war, die Rachenhinterwand nicht erreichte und höchstens ein ipsilateraler suspekter Lymphknoten vorlag.

Der primäre Endpunkt der Studie war das getrennt auszuwertende 2‑Jahres-Gesamtüberleben, für das in beiden Armen im Vergleich zu historischen Kontrollen ein Wert von 94 % postuliert wurde. Sekundäre Endpunkte waren das progressionsfreie Überleben (PFS), die vom Patienten berichtete Symptomlast und die Lebensqualität, Toxizität nach CTCAE v4, Magensondenrate nach einem Jahr sowie der Ernährungsstatus nach *Functional Oral Intake Scale.*

Eine vordefinierte Interimsanalyse nach insgesamt 70 rekrutierten PatientInnen forderte den Abbruch der Studie, sofern in einem Studienarm mehr als 5 % (2/35) therapieassoziierte Todesfälle vorliegen sollten.

## Ergebnisse

Die Studie rekrutierte 61 PatientInnen, von denen 30 in den RT-Arm und 31 in den OP-Arm randomisiert wurden. Die Patientencharakteristika waren zwischen beiden Armen ausgeglichen. Im OP-Arm erhielten 78 % (21/31) der PatientInnen eine adjuvante Radiotherapie, alle ohne simultane Chemosensibilisierung. Bei 33 % (9/31) lag ein positiver oder knapper Resektionsrand vor. Im RT-Arm erhielten 72 % (21/30) der PatientInnen eine simultane Chemosensibilisierung. Die Studie wurde aufgrund der dokumentierten Toxizitäten im OP-Arm vorzeitig abgebrochen: Hier traten zwei therapieassoziierte Todesfälle (Blutung am vierten postoperativen Tag und zervikale Osteomyelitis 110 Tage nach OP) sowie ein weiterer, nicht therapieassoziierter Todesfall (Myokardinfarkt) auf. Zusätzlich wurde im OP-Arm ein Lokalrezidiv diagnostiziert. Im RT-Arm gab es im Nachbeobachtungszeitraum weder Todesfälle noch Rezidive. Der primäre Endpunkt des 2‑Jahres-Gesamtüberlebens wurde aufgrund der kurzen Nachbeobachtungszeit mit median 17 Monaten noch nicht berichtet.

Eine höhergradige Toxizität (≥ Grad 2) lag im RT-Arm bei 67 % (2/30) und im OP-Arm bei 71 % (22/31) der PatientInnen vor, was keinen statistisch signifikanten Unterschied darstellte. Signifikant mehr PatientInnen erfuhren im RT-Arm eine Dysgeusie (*p* = 0,03) oder Anorexie (*p* = 0,01) ≥ Grad 2. Gemäß *Functional Oral Intake Scale* hatten 81 % (13/16) der PatientInnen im RT-Arm gegenüber 38 % (5/13) im OP-Arm (*p* = 0,05) keinerlei funktionelle Einschränkungen bei der Nahrungsaufnahme. Eine Magensonde war nach einem Jahr in keinem Fall nötig. Der Serumkreatininwert war zum Zeitpunkt der letzten Nachsorge im RT-Arm signifikant schlechter als im OP-Arm (*p* = 0,04), ein Unterschied bezüglich der Inzidenz eines Nierenversagens wurde jedoch nicht berichtet.

Die patientenberichteten Endpunkte unterschieden sich nicht wesentlich zwischen beiden Behandlungsarmen. Die Schluckfunktion nach *MD Anderson Dysphagia Inventory *(MDADI) unterschied sich nicht signifikant. Die einzig explorativ statistisch signifikanten Unterschiede bestanden bezüglich Husten und Gewichtsverlust nach EORTC QLQ-H&N35 zugunsten des RT-Arms.

## Schlussfolgerungen der AutorInnen

Die primäre transorale Operation HPV-assoziierter Oropharynxkarzinome war in dieser Studie mit einem inakzeptabel hohen Risiko für Grad-5-Toxizität verbunden und sollte zurückhaltend eingesetzt werden.

## Kommentar

Die Therapiedeeskalation wird beim HPV-assoziierten Oropharynxkarzinom aktuell in zahlreichen Studien mit unterschiedlichen Ansätzen untersucht. Zu diesen Ansätzen zählen unter anderen i.) eine Modifikation bzw. ein Verzicht der Chemosensibilisierung bei Radiotherapie, ii.) eine Modifikation der Dosis und/oder des Volumens der Radiotherapie oder iii.) der Einsatz weniger invasiver transoraler Operationsverfahren, ggf. mit risikoadaptierter Adjuvanz [1, 2].

Die randomisierte NRGHN002-Studie untersuchte beispielsweise den Verzicht einer Chemosensibilisierung mit Cisplatin bei PatientInnen mit HPV-assoziiertem Oropharynxkarzinom (T1‑3 N1–2b oder T3 N0 [TNMv7]) bei primärer Radio(chemo)therapie bis 60 Gy [3]. Insgesamt wurden hier 306 PatientInnen randomisiert. Der präspezifizierte Endpunkt des PFS wurde bzgl. der Nichtunterlegenheit zu historischen Kontrollen lediglich im Arm mit Chemosensibilisierung (2-J-PFS 90,5 %) erreicht. Hieraus wurde das Studiendesign der randomisierten Phase-III-Studie NRGHN005 abgeleitet, die aktuell rekrutiert und eine primäre cisplatinbasierte Radiochemotherapie bis 60 Gy bzw. bis 70 Gy untersucht.

Einen anderen Ansatz verfolgte die E3311-Studie, die eine risikoadaptierte Adjuvanz nach TOS bei HPV-assoziiertem Oropharynxkarzinom überprüfte [4]. PatientInnen mit niedrigem Risiko (R0, N0–1 [TNMv7], ECE−) erhielten keine Adjuvanz, während PatientInnen mit hohem Risiko (R1/2, ECE > 1 mm, > 5 pos. LK) eine adjuvante cisplatinbasierte Radiochemotherapie bis 66 Gy erhielten. Bei PatientInnen im intermediären Risikoprofil (knappe Ränder, 2–4 pos. LK, Pn1, LV1, ECE ≤ 1 mm) erfolgte eine Randomisierung zu einer adjuvanten Radiotherapie bis 50 Gy bzw. 60 Gy. Das 2‑J-PFS der randomisierten Arme mit insgesamt 208 randomisierten PatientInnen lag bei 94,9 % nach 50 Gy und 96 % nach 60 Gy. Auch der dosisreduzierte Arm erfüllte damit die präspezifizierte Schwelle der Nichtunterlegenheit.

Bislang bestanden jedoch keine Daten bezüglich eines Vergleichs eines primär strahlentherapeutischen gegenüber eines primär operativen Vorgehens in der Therapiedeeskalation des HPV-assoziierten Oropharynxkarzinoms. Diesen randomisierten Vergleich beschreibt nun die dargestellte ORATOR2-Studie, deren jeweilige Studienarme auf der NRGHN002- bzw. E3311-Studie basieren. Bei dieser Studie sind drei Punkte besonders zu diskutieren:

Erstens der vorzeitige Studienabbruch aufgrund zweier therapieassoziierter Todesfälle im OP-Arm. Bei einem Todesfall trat eine fatale Blutung auf, obwohl protokollgemäß sowohl ein protektives Tracheostoma als auch eine Gefäßligatur durchgeführt wurden. Ebenfalls bestanden für die Teilnahme an der Studie konkrete Anforderungen an die chirurgische Expertise der teilnehmenden Zentren, die sich eng an die Anforderungen der E3311-Studie anlehnten [5]. Bei dem zweiten Todesfall führte eine zervikale Osteomyelitis erst am 110. postoperativen Tag und nach adjuvanter Therapie zum Tod. Nach 31 im OP-Arm behandelten PatientInnen lag damit eine Grad-5-Toxizität von 6,5 % (2/31) vor. Auch wenn dadurch die präspezifizierte Abbruchregel der Studie aktiviert wurde, ist diese hohe Rate schwer einzuordnen. In der ersten ORATOR-Studie, die eine primäre Radio(chemo)therapie mit primärer Operation mittels TOS +/− Adjuvanz in „konventioneller“ Intensität beim Oropharynxkarzinom randomisiert untersuchte, trat ebenfalls eine fatale Blutung auf (2,9 %; 1/34) [6, 7]. Demgegenüber berichtete eine Analyse der SEER-Datenbank von knapp 5000 PatientInnen, die eine TOS bei Oropharynxkarzinom erhielten, eine 30-Tages-Letalität von 1 % und eine 90-Tages-Letalität von 1,7 % [8]. Möglicherweise ist daher die 30- bzw. 90-Tages-Letalität ein zu knappes Zeitfenster, um das Letalitätsrisiko zu beurteilen. Zusätzlich könnte die Patientenselektion in nichtrandomisierten Daten zu günstigeren Ergebnissen der TOS beitragen [9]. Schlussendlich beurteilen die AutorInnen der ORATOR2-Studie die hohe Rate an Grad-5-Toxizität durchaus als mögliches Zufallsergebnis. Dennoch sei die Rate im Kontext des Angebots einer potenziellen Therapiedeeskalation nicht zu rechtfertigen, was den Studienabbruch aus ihrer Sicht erforderte.

Zweitens ist das exzellente PFS nach primärer Radio(chemo)therapie festzuhalten. Im bisherigen medianen Follow-up von 17 Monaten trat im RT-Arm kein Rezidiv oder Todesfall auf, was aktuell in einem PFS von 100 % resultiert. In der ORATOR-Studie lag das PFS für PatientInnen mit p16-positiven Tumoren 3 Jahre nach Therapie bei 93,3 % nach Radio(chemo)therapie vs. 90,0 % nach TOS [10]. Diese Ergebnisse sind bezüglich der Effektivität kaum zu verbessern, sodass der Optimierung der therapiebedingten Morbidität und Mortalität eine Schlüsselrolle zukommt.

Drittens zeigte sich in der explorativen Analyse der Schluckfunktion nach MDADI kein signifikanter Unterschied zwischen beiden Studienarmen. Dies steht im Widerspruch zur ersten ORATOR-Studie, die trotz höherer Gesamtdosis der Strahlentherapie eine signifikant bessere Schluckfunktion im RT-Arm gegenüber dem OP-Arm zeigte, allerdings ohne die präspezifizierte Schwelle eines klinisch bedeutsamen Unterschieds zu überschreiten. Auch in der E3311-Studie und der NRGHN002-Studie zeigten sich bzgl. Dosisreduktion der Radiotherapie und Verzicht auf Cisplatin keine klaren Unterschiede bzgl. der Lebensqualität und Schluckfunktion. Daher bleibt die Erhebung von „patient-reported outcomes“ zur letztlichen Demonstration einer verbesserten Lebensqualität in Deeskalationskonzepten hochgradig relevant. Nichtsdestotrotz widerlegt auch die ORATOR2-Studie die initial postulierte These, dass eine primäre TOS zu einer besseren Schluckfunktion führen würde im Vergleich zu etablierten Therapiealternativen [11].

### Fazit

Zusammengefasst liefert die zweite Studie mit einem randomisierten Vergleich zwischen primärer Radio(chemo)therapie und primärer TOS trotz des frühzeitigen Studienabbruchs aufgrund der Grad-5-Toxizität im OP-Arm wertvolle Daten. Das aktuelle PFS ist im RT-Arm mit 100 % nach 17-monatigem vorläufigem Follow-up exzellent und vielversprechend. Dennoch bleibt festzuhalten, dass eine Therapiedeeskalation beim HPV-assoziierten Oropharynxkarzinom (noch) kein Therapiestandard ist und dafür Langzeitdaten aus Phase-III-Studien unerlässlich sind. Bei – nach allem, was wir wissen – vermutlich gleichwertigen Therapieoptionen sollten alle PatientInnen mit HPV-assoziierten Oropharynxkarzinom von strahlentherapeutischer Seite über die Option einer primären Radio(chemo)therapie aufgeklärt werden.

*Alexander Fabian, Nils H. Nicolay und David Krug, Freiburg und Kiel*

## Abkürzungen

ECE: Extrakapsuläres Wachstum (*„extracapsular extension“*)
MDADI: *MD Anderson Dysphagia Inventory*
PFS: Progressionsfreies Überleben (*„progression-free survival“*)
TOS: Transorale Chirurgie (*transoral surgery*)
